# Case report: Ventriculoperitoneal shunt disconnection resulting in migration of the distal catheter entirely into the abdominal cavity due to seizure

**DOI:** 10.3389/fsurg.2022.1012720

**Published:** 2022-09-23

**Authors:** Xiang Yang, Chaohua Yang, Yuekang Zhang

**Affiliations:** Department of Neurosurgery, West China Hospital of Sichuan University, Chengdu, China

**Keywords:** ventriculoperitoneal (VP), disconnection, catheter, seizure, valve

## Abstract

Ventriculoperitoneal (VP) shunt disconnection, a VP shunt complication, can be caused by several factors. We report the case of a young man who suffered VP shunt disconnection, and whose entire distal catheter migrated into the abdominal cavity due to a seizure. To our knowledge, risk factors for seizures related to shunt disconnection have not been previously evaluated. We report this rare case to highlight the fact that seizures are not negligible in increasing the probability of disconnection and migration of the entire distal catheter into the abdominal cavity, and the standardized treatment of traumatic seizures is extremely important.

## Introduction

Ventriculoperitoneal (VP) shunt placement is one of the most efficient methods for the treatment of hydrocephalus; it is the third most commonly performed procedure during neurosurgical residency (1). However, complications following VP shunt placement are not uncommon, which can cause harm to patients and further their economic burden (2–6). Shunt disconnection is defined as discontinuity between the catheter and valve, which can be caused by promoting shear stress on the catheter, including fractures resulting from biomechanical stress as the patient grows, degradation of the catheter itself, stiffness of the catheter, and scar tissue formation (3). Poor knot tying is also a factor affecting shunt disconnection between the valve and catheter (3, 7). However, the risk factors of seizures related to shunt disconnection have not been previously evaluated, which is also a non-negligible accomplice. Herein, we report a rare case of VP disconnection resulting in migration of the distal catheter into the abdominal cavity entirely due to an epileptic seizure. Our case enhances the understanding of the risk factors for seizures associated with VP disconnection and contributes to improving the proper management of such patients. This paper has been written in agreement with the CARE guidelines.

## Case presentation

### Presentation and examination

A 25-year-old man visited our emergency department because of a progressive disorder of consciousness within 3 days. Four months before admission, the patient underwent a right large decompressive craniectomy due to a traffic accident at a local hospital. The patient recovered well after the surgery and was successfully discharged. However, he experienced an epileptic seizure 2 months later, and cranial computed tomography (CT) revealed obstructive hydrocephalus. Subsequently, the patient received a left VP shunt at the local hospital with a good outcome, except for paroxysmal seizures because of substandard treatment of epilepsy. He was administered sodium valproate orally to control his epileptic seizures after VP shunt, but it did not work well. The patient and his family did not actively seek specialist help to better control his seizures.

On physical examination at admission, the patient was in a light coma with extremely high scalp tension in the decompression bone window. Bilateral pupil reflex to light disappeared. The rebound of the shunt reservoir was not abnormal, and there was no subcutaneous swelling behind the ear. It was difficult to track the entire distal shunt catheter under the subcutaneous tunnel.

### Imaging findings

**Figure 1:**
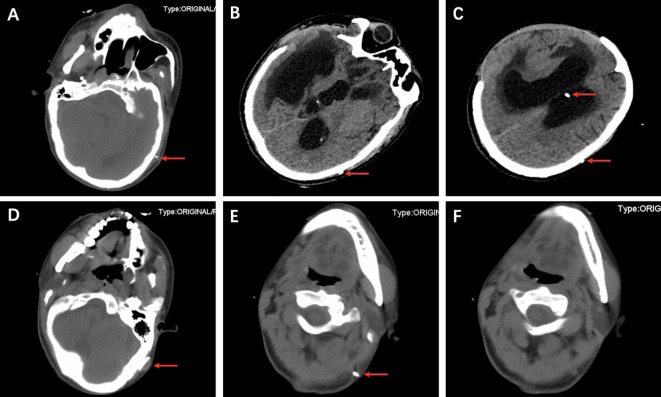
CT showing obvious ventriculomegaly association with extravasation of CSF and the ventricular end of the catheter placed in the correct position (red arrows) (**A–C**). CT shows the valve (**D**, red arrow) and the metal connector (red arrows) (**E**). CT shows the distal catheter disappearing at the joint (**F**).

**Figure 2:**
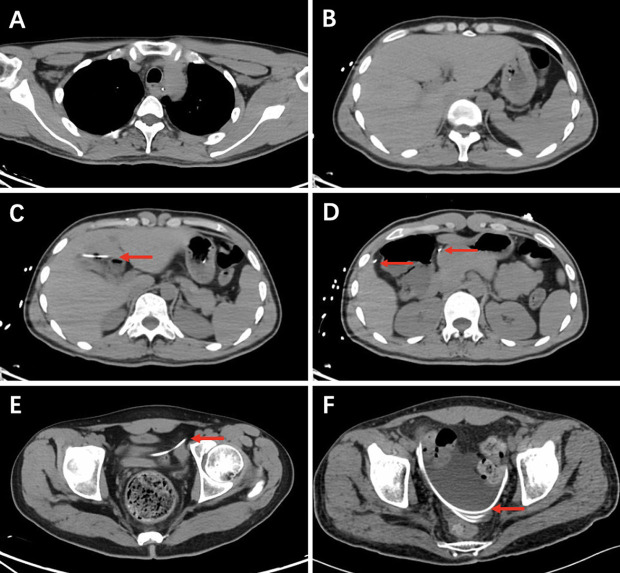
Chest and whole abdomen CT showing no subcutaneous shunt catheter (**A,B**). CT shows the distal catheter placed entirely in the abdominal cavity without knotting (red arrows) (**C–E**). Postoperative CT shows the reserved distal catheter placed in the pelvic cavity accompanied by CSF drainage (red arrows) (**F**).

**Figure 3:**
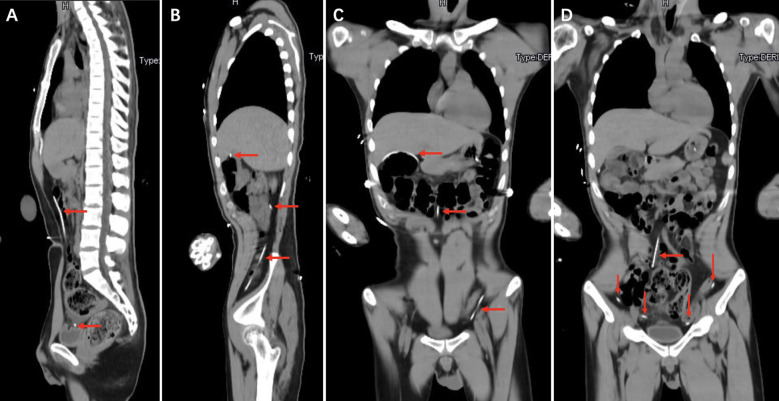
Chest and whole abdomen computed tomography showing the distal catheter placed entirely in the abdominal cavity without knotting (red arrows).

On the cranial CT scan, obvious ventriculomegaly associated with extravasation of cerebrospinal fluid (CSF) was observed, and the ventricular end of the catheter was in the correct position (Figures 1A–C). CT of the head, neck, chest, and whole abdomen showed no subcutaneous shunt catheter from the valve to the abdominal incision, and the distal shunt catheter was placed in the abdominal cavity entirely without knotting formation (Figures 1D–F, 2A–E, 3).

### Surgical procedure

Exploratory surgery revealed that the VP shunt was completely disconnected between the valve and distal catheter, and the distal catheter completely disappeared in both the old retroauricular and abdominal incisions. The valve outlet was completely blocked, and clear CSF spurted out after removing the shunt valve. Therefore, a ventricular catheter was inserted. Considering the softness of the shunt, the valve and distal catheter were replaced by another device. A new abdominal incision was made at the distal end of the catheter to reduce the chance of infection. To minimize surgical trauma, the distal catheter was not removed laparoscopically.

### Postoperative course

**Figure 4:**
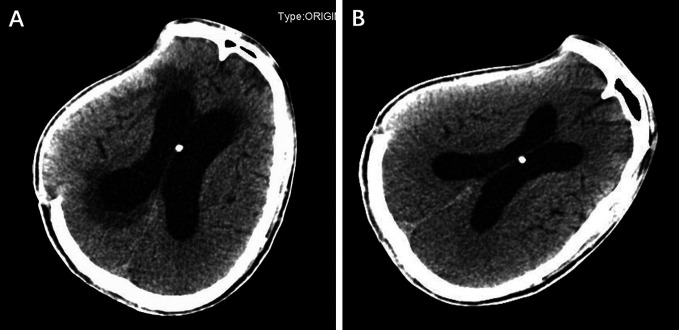
The cranial computed tomography examinations on the 5th (**A**) and 20th (**B**) postoperative days confirmed that the ventricle system was gradually narrowed.

**Figure 5:**
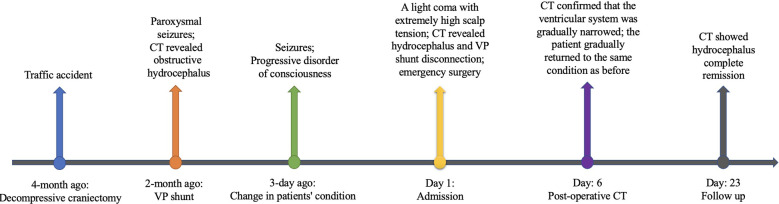
Timeline of the symptoms, management, and outcomes of the patient.

After surgery, several cranial CT examinations confirmed that the ventricular system was gradually narrowed (Figure 4), and the scalp in the decompression bone window collapsed at follow-up. Postoperative whole-abdominal CT showed the reserved distal catheter placed in the pelvic cavity accompanied by CSF drainage (Figure 2F). The patient was administered levetiracetam orally to control his seizures, and he gradually returned to the same condition as before. The timeline of the symptoms, management, and outcomes of the patient are summarized in Figure 5.

## Discussion

VP shunt surgery is one of the most established strategies to treat hydrocephalus by diverting the accumulation of CSF from the cerebral ventricles to the abdominal cavity (8). Although VP surgery has a long history, it is associated with a variety of adverse events, including shunt obstruction, mechanical failure (fracture, disconnection, migration, subcutaneous extrusion, or protrusion of the catheter), infection, intracranial hemorrhage, and intracranial hypotension (4, 9–11). Most patients with complications require additional surgery, resulting in a significant economic burden on the healthcare system, and some patients have extremely poor outcomes due to unfortunate complications (6, 12, 13).

Shunt disconnection is defined as a discontinuity between the catheter and the valve. It is a relatively high cause of failure after shunt obstruction, and the main sticking point is the presence of a connector (4); however, previous literature has not accurately reported distal catheter migration entirely into the abdominal cavity, especially when the seizure is thought to be the culprit. We report this rare case to foster an understanding of the risk factors of seizures associated with VP disconnection and to provide better management of such patients.

Given that the valves typically attach to the subcutaneous tissue behind the ear, as well as the developed muscle groups in the neck, it is obvious that the weak connectors are most likely to be attacked by persistent and repeated neck muscle contractions in the event of epileptic seizures. The literature has highlighted the close link between shunt disconnection and poor knot tying (3), but it cannot withstand the powerful force of the constant tugging that can occur during epileptic seizures, even with hard knot tying. In this case, a single epileptic fit was enough to cause disconnection; however, a good learning point regards the higher risk of such disconnection happening in patients who failed to control the seizures and evolve into status epilepticus, hence requiring admission to the neuro-intensive care unit (14).

Another disconnection factor of the mechanical properties of valves and catheters should also be included, as the standard catheter for each device has different mechanical properties in terms of elasticity and pliability (4). Research has shown that the increased stiffness of the device may cause inadequate application of a suture tie used to secure the catheter to the valve, that is, a less stiff catheter and valve can reduce disconnection (4). In our report, the patient was given a device for the first VP shunt surgery, whose connector is metal with a relatively stiff catheter, which increases the probability of disconnection during epileptic seizures. Therefore, another device with less stiffness was chosen for the second VP operation.

One of the questions that puzzled us was how to explain the distal catheter migration entirely into the abdominal cavity. Abdominal complications following a VP shunt included a pseudocyst formation, an obstruction, or shunt disconnection and migration (15). Abdominal adhesion can favor VP shunt migration but is commonly caused by bowel perforation. In some cases, the abdominal adhesion can promote the formation of CSF pseudocysts because it interferes with the absorption of CSF in the abdominal cavity, and the distal catheter is usually coiled in the pseudocyst (15–17). Neither perforation nor pseudocyst was observed in our case on CT scan, and simple intra-abdominal adhesions were not sufficient to completely drag the catheter into the abdominal cavity, relying on gastrointestinal peristalsis (5, 18). Moreover, the patient had no history of abdominal surgery or trauma and no evidence of abdominal adhesions. Knot formation on the distal catheter has previously been reported in some rare cases (18–20), which can trap the terminal end of the peritoneal catheter or be stuck to drag the catheter entirely into the abdominal cavity. However, the patient in our case did not experience knotting formation on the entire abdominal CT scan. Therefore, the only plausible explanation is that seizures increase contractions of the abdominal muscles and smooth muscles of the gut that pull the distal catheter completely into the abdominal cavity.

## Conclusion

Many factors affect VP shunt disconnection between the valve and catheter, and poor knot tying and stiff devices may contribute to it. However, seizures, although not previously reported, are also non-negligible accomplices. Therefore, the standardized treatment of traumatic seizures is extremely important.

## Data Availability

The original contributions presented in the study are included in the article, further inquiries can be directed to the corresponding author.
